# Differential gene expression of salt-tolerant alfalfa in response to salinity and inoculation by *Ensifer meliloti*

**DOI:** 10.1186/s12870-024-05337-5

**Published:** 2024-07-06

**Authors:** Seth Lundell, Bill Biligetu

**Affiliations:** Department of Plant Sciences, College of Agriculture and Bio-Resources, University of Saskatchewan, 51 Campus Dr., Saskatoon, SK S7N5A8 Canada

**Keywords:** Rhisobium and genotype interaction, Salt tolerant DEG, Recurrent selection

## Abstract

**Background:**

Alfalfa (*Medicago sativa* L.) experiences many negative effects under salinity stress, which may be mediated by recurrent selection*. *Salt-tolerant alfalfa may display unique adaptations in association with rhizobium under salt stress.

**Results:**

To elucidate inoculation effects on salt-tolerant alfalfa under salt stress, this study leveraged a salt-tolerant alfalfa population selected through two cycles of recurrent selection under high salt stress. After experiencing 120-day salt stress, mRNA was extracted from 8 random genotypes either grown in 0 or 8 dS/m salt stress with or without inoculation by *Ensifer meliloti*. Results showed 320 and 176 differentially expressed genes (DEGs) modulated in response to salinity stress or inoculation x salinity stress, respectively. Notable results in plants under 8 dS/m stress included upregulation of a key gene involved in the Target of Rapamycin (TOR) signaling pathway with a concomitant decrease in expression of the SNrK pathway. Inoculation of salt-stressed plants stimulated increased transcription of a sulfate-uptake gene as well as upregulation of the Lysine-27-trimethyltransferase (EZH2), Histone 3 (H3), and argonaute (AGO, a component of miRISC silencing complexes) genes related to epigenetic and post-transcriptional gene control.

**Conclusions:**

Salt-tolerant alfalfa may benefit from improved activity of TOR and decreased activity of SNrK1 in salt stress, while inoculation by rhizobiumstimulates production of sulfate uptake- and other unique genes.

**Supplementary Information:**

The online version contains supplementary material available at 10.1186/s12870-024-05337-5.

## Background

One of the greatest impacts of salinity stress on legumes, including alfalfa (*Medicago sativa* L.)*,* is the abortion of nodulation by beneficial nitrogen fixing bacteria (i.e., *Ensifer meliloti*). Alfalfa associates with rhizobium such as *E. meliloti* in a mutualistic relationship, which involves the exchange of photosynthetically-derived sugars from the plant for biologically-fixed nitrogen from the bacteroid. Under non-stressful growth conditions, this exchange is mutually beneficial. However, in periods of environmental stress, such as salinity stress, the bacteroids may transit from being net-assets to net-parasites as they drain the plant of carbohydrates [1]. Under these conditions, the host plant will commonly terminate the relationship as it attempts to save valuable energy resources for its own usage, rather than exchanging them for fixed nitrogen [1–3].

A number of studies have been conducted to increase the salinity tolerance of the alfalfa-rhizobium complex by inoculating alfalfa with salt-tolerant bacteria [4–6]. While inoculation with salt-tolerant bacteria often provides benefits such as increased yield and vigorous plant growth, the superior salt tolerance of *E. meliloti* relative to alfalfa [7] suggests that it may be a yet-more effective approach to inoculate salt-tolerant alfalfa with *E. meliloti* to promote plant fitness under saline conditions.

The findings of previous transcriptomic studies over the past decade have revealed many stress-related genes in alfalfa. RNA-seq studies examining salt-tolerant vs susceptible cultivars have revealed that tolerant genotypes can display increased transcription for genes related to ROS scavenging, Ca^2+^ signaling (including the Salt Overly Sensitive (SOS1, SOS2, & SOS3) series of pathways), as well as High affinity Potassium Transporters (HKT) genes [8–12]. The complexity of these effects would most likely be compounded by inoculation and breeding, as inoculants themselves can stimulate up- or down regulation of genes [13], and genetic improvement can cause allelic shifts. Despite these interactions, few studies have examined the combination effects of selection and inoculation in salt-stressed alfalfa.

To address this knowledge gap, we conducted an experiment whereby a salt tolerant population of alfalfa was grown in non-saline (0 dS/m E.C.) or moderately (8 dS/m E.C.) saline conditions, with or without inoculation by *E. meliloti*. We predicted that inoculation would stimulate transcription of genes related to nitrogen metabolism, potentially related to bacteroid sourced nitrogen, as well as stress-related genes responsible for producing ROS scavengers, osmoprotectants, calcium signaling, etc. The objective of this project was thus to determine the gene expression profile of salt-tolerant alfalfa in response to inoculation by the rhizobium *E. meliloti* under 8 dS/m E.C. salinity stress.

## Methods

### Experimental design, plant & bacterial materials

The experiment, and breeding history of alfalfa populations, are described in our previous publication [14]. Briefly, a split-plot experiment with three treatments (rhizobium, three generations of alfalfa populations, and three salinity levels) was implemented. The bacteria used were *Ensifer meliloti* strain rm1021. Three alfalfa populations were developed through three cycles (Generations) of recurrent selection for salt tolerance. This experiment extracted RNA from Generation 2 (G2) population (the best performing alfalfa generation based on biomass, chlorophyll content, and osmoprotectant production), which originated from the 2nd cycle of selection at 20 dS/m E.C. salinity stress. Leaf samples were collected at 120 days from 0 and 8 dS/m treated G2 plants with or without rhizobium (*E. meliloti*) inoculation. Each sample taken was replicated twice for a total of 8 samples (1 alfalfa population × 2 rhizobium treatments × 2 salinity levels × 2 replicates).

### RNA isolation, library preparation and sequencing

Samples were lyophilized in liquid nitrogen before being stored in a -80 °C freezer until analysis. A Qiagen RNeasy Plant mini kit (Qiagen Gmbh, Hilden, Germany) was used to extract RNA as per the manufacturer’s instructions. Briefly, plant tissues were ground in liquid nitrogen and homogenized with a QIAshredder during centrifugation. Ethanol was added to bind with RNA. The resulting solid was washed 3 × in the buffers provided, and the RNA was eluted in RNA free water. Quality of samples was determined through 260/280 nm readings on a Thermofisher NanoDrop 8000 (Waltham, MA, USA). After collection, 40 uL of resulting suspended mRNA from each sample was sent on ice to Novogene (California, USA) for library preparation and sequencing on an Illumina Novaseq 6000 platform (Illumina, San Diego CA, USA).

### Mapping and differential expression analysis

Raw reads were cleaned of adapters using Trimmomatic v.0.36 software [15], with threads = 4, phred33, and default quality cut-off values. The quality of resulting files was checked with FASTQC software [16]. After checking file quality, reads were moved through the bioinformatics pipeline in the Digital Research alliance of Canada for most initial steps, with differential expression being calculated in R. Once prepared, trimmed reads were matched to the reference genome prepared by a previous work [17] (available at 10.6084/m9.figshare.12327602.v3) [18] using STAR (v2.6.1a)[19] from within the quantification software ‘RSEM’ with the –star flag (parameters from https://github.com/ENCODE-DCC/long-rna-seq-pipeline/blob/master/DAC/STAR_RSEM.sh) [20]. From the same code, read counts were conducted using the software ‘RSEM’ [21]. ‘isoform.results’ files were passed on to the differential expression analysis software ‘DESeq2’ [22] in R version 3.6.3 [23]. ‘DESeq2’ was deemed suitable for the dataset as it is capable of defining differentially expressed genes from experiments with 2–3 replicates by accounting for the high ‘noise’ of background expression levels through shrinking the dispersion values to an average fitted value [22]. Through this conservative approach, genes with very low dispersion values are fitted with a higher value to prevent false-positives, while values with dispersion values > 2 standard deviations above the fitted mean are treated ‘as-is’ also to prevent false positives. To further reduce the risk of false positives and discover significant DEGs, we identified DEGs using a Log fold change (Log_2_FC) > 2 and a stringent false discovery rate (FDR) of *P* < 0.001, an FDR which either matched or exceeded the stringency of many previous studies [8–12, 24–26]. *P* values were adjusted in DESeq2 using the Benjamini and Hochberg method. Significant DEGs were calculated separately for both salinity (8 vs 0 dS/m) and inoculant (inoculated vs non-inoculated) treatments as well as an interaction term (inoculated plants in 8 dS/m vs non-inoculated plants in 8 dS/m).

### Functional annotation and analysis

Functional annotations of genes were retrieved for significant DEGs from the data frames prepared in association with the reference genome provided by a previous work [27]. These included Gene Ontology (GO) terms, KEGG Orthology (KO) terms, and a BLASTp search against NCBIs Non-Redundant (NR) protein database. KAAS analysis was also conducted with DEG associated protein sequences (also supplied by the previous work [27]) to elucidate relationships between DEGs in KEGG metabolic pathways. Furthermore, gene Ontology (GO) and Kyoto Encyclopedia of Genes and Genomes (KEGG) enrichment tests were conducted. GO enrichment analyses were conducted using the R package ‘topgo’ [28]; GO terms were tested using Fishers exact test which utilizes a hypergeometric distribution, and were considered enriched with a *P* = 0.05. KEGG enrichment was also done in R with a hypergeometric calculation and with significance determined at *P* = 0.05.

## Results

### High throughput sequencing and assembly

Illumina sequencing generated total 173,023,874 paired end (PE) raw reads for the 8 samples sequenced. Of the reads, 171,165,017 (98.9%) remained after trimming, and 94.91% of trimmed reads were mapped to the alfalfa reference genome using STAR (v2.7.9). All samples showed a high percentage of mapping to the alfalfa reference genome, ranging from 91.96% to 95.97% of reads mapped. There were a high percentage of multi-mapped reads. For information regarding raw and trimmed read counts and lengths, refer to Table 1.

**Table 1:** Summary of Illumina sequencing data and mapped read data for the assayed alfalfa samples. Samples are given in Replicate-bacterial treatment (Control vs *Ensifer meliloti*). Salinity level (0 vs 8 dS/m)

Treatment	Total Reads	Uniquely mapped reads	Multi-mapped reads	Total mapped reads	Mapping rate (%)	Average Mapped Length
R5-Cont.0	21,036,054	7,994,126	12,096,115	20,090,241	95.5	294.16
R4-Cont.0	20,482,260	7,782,229	11,053,691	18,835,920	92.0	293.88
R6-Cont.8	21,060,149	7,872,397	12,271,725	20,144,122	95.7	293.56
R5-Cont.8	22,760,905	8,668,412	13,010,971	21,679,383	95.3	294.51
R5-Ensif.0	21,206,181	7,879,943	11,972,722	19,852,665	93.6	294.01
R4-Ensif.0	22,664,318	9,003,802	12,747,509	21,751,311	96.0	294.48
R5-Ensif.8	21,230,232	8,089,400	12,236,557	20,325,957	95.7	294.52
R4-Ensif.8	20,724,918	7,738,954	12,035,028	19,773,982	95.4	294.24

### Identification of DEGs

**Fig. 1:**
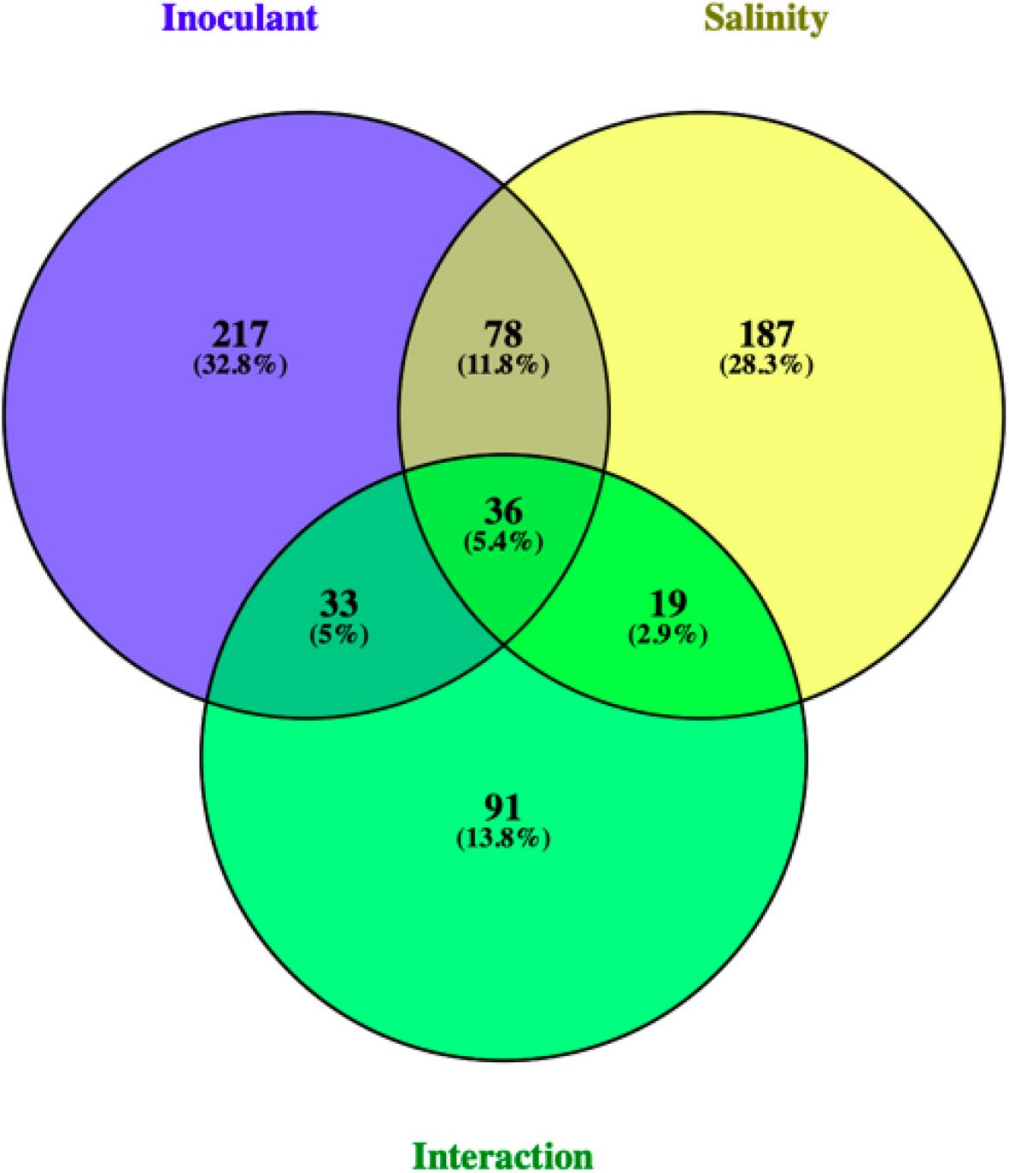
Venn diagram for number of overlapping DEGs in leaves of salt tolerant alfalfa affected by rhizobium inoculation, salinity, and salinity x rhizobium inoculation

Between all treatments, there were many up- and down-regulated DEGs identified after mapping to the alfalfa reference genome. In total, there were 495 significant genes including 364 rhizobia-related genes, 320 salinity-related genes, and 179 interaction- (salinity x rhizobium) related genes. Specifically, rhizobium inoculated plants displayed 173 (47.5%) upregulated and 191 (52.5%) downregulated genes relative to non-inoculated plants. Salinity treated plants displayed 158 (49.4%) upregulated and 162 (50.6%) down-regulated genes compared to control plants. Inoculated, salt-stressed plants displayed 76 (42.5%) up-regulated and 103 (57.5%) down-regulated genes relative to non-inoculated, salt-stressed plants (Fig. 1).

### Functional annotation of DEGs

#### GO enrichment

**Fig. 2:**
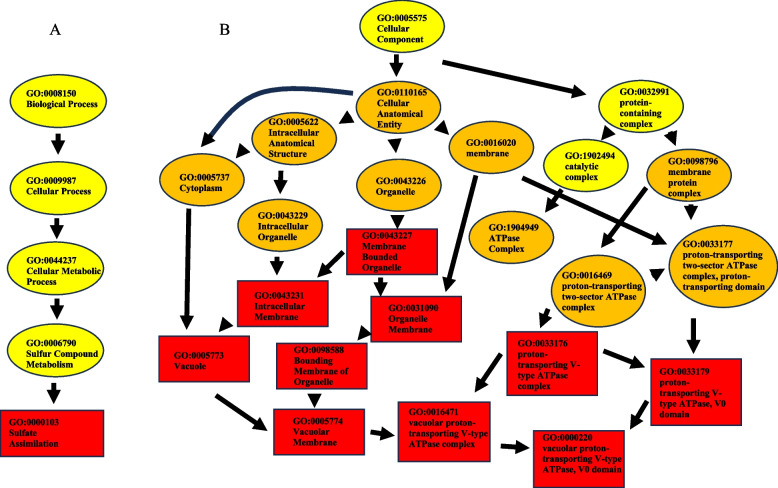
Gene ontology (GO) analysis of differentially expressed genes (DEGs) in leaf tissues of salt tolerant alfalfa: **A **Biological Process GO graph of enriched terms annotated to inoculation x salinity DEGs; **B **Cellular Component GO graph annotated to salinity DEGs. *P* values and the number of significant/total annotations given in each box. Color of boxes represents most to least significant from Red–orange-yellow. Enrichment based on Fishers exact test

Of the total 495 significant DEGs, 251 salinity, 260 rhizobium, and 136 of their interaction were annotated into the three GO categories: Molecular Function (MF), Biological Process (BP), and Cellular Component (CC). In addition to this information, the proportion of total DEGs assigned to a GO category are given in Table 2. Significant candidate genes annotated to GO terms are also given in Table 3. There were a total of 12 enriched GO terms across BP and CC categories. There were no enriched terms within the MF category. Within BP, there were two significant GO terms; GO:0001505 (regulation of neurotransmitters) and GO:0000103 (sulfate assimilation, refer to Fig. 2A). Within salinity-related DEGs, GO:0001505 was annotated to two significant DEGs (MS.gene21474.t1, MS.gene012657.t1), while within the salinity x rhizobium interaction, GO:0000103 was annotated to 1 gene (MS.gene006381, see Table 3). Of salinity-related DEG in the CC category, there were 10 enriched GO terms (see Table 4, refer to Fig. 2B).

**Table 2:** Total number of DEGs as well as the number and percent annotated with GO categories

Treatment Pool	Category	GO Annotated DEGs	Total DEGs	Percent of Total
Salinity (S)	MF	226	320	70.6
	BP	15	320	4.7
	CC	10	320	3.1
Inoculant (I)	MF	230	364	63.2
	BP	14	364	3.8
	CC	16	364	4.4
S x I Interaction	MF	126	179	70.4
	BP	5	179	2.8
	CC	5	179	2.8

*MF* Molecular Function, *BP* Biological Processes, *CC* Cellular Component

**Table 3:** List of candidate genes associated with the enriched GO terms, as well as KEGG or Nr annotations (if present)

Gene	GO	DEG Pool	KEGG	Nr ID	GO Term
MS.gene21474.t1	BP	Salinity	K02437	XP_003611464.1	GO:0001505
MS.gene012657.t1	BP	Salinity		XP_003610457.2	GO:0001505
MS.gene006381.t1	BP	Interaction		XP_013469727.1	GO:0000103
MS.gene067465.t1	CC	Salinity		KEH39627.1	GO:0098588:GO:0031090:GO:0043227:GO:0043231
MS.gene50737.t1	CC	Salinity	K02154	XP_003607000.1	GO:0098588GO:0000220GO:0033179GO:0031090GO:0005773 GO:0005774GO:0016471GO:0033176GO:0043227GO:0043231

Nr ID is the protein accession number in NCBI non-redundant protein database

*BP* Biological Processes, *CC* Cellular Component

**Table 4:** List of significantly enriched GO terms in the Cellular Component category within the salinity pool of differentially expressed genes

GO ID	Term	Total Annotated DEG	Significant DEGs	Expected DEGs	P-value
GO:0098588	bounding membrane of organelle	40	2	0.2	0.016
GO:0000220	vacuolar proton-transporting V-type ATPase, V0 domain	5	1	0.03	0.025
GO:0033179	proton-transporting V-type ATPase, V0 domain	5	1	0.03	0.025
GO:0031090	organelle membrane	52	2	0.26	0.027
GO:0005773	vacuole	9	1	0.05	0.045
GO:0005774	vacuolar membrane	9	1	0.05	0.045
GO:0016471	vacuolar proton-transporting V-type ATPase complex	9	1	0.05	0.045
GO:0033176	proton-transporting V-type ATPase complex	9	1	0.05	0.045
GO:0043227	membrane-bounded organelle	71	2	0.36	0.047
GO:0043231	intracellular membrane-bounded organelle	71	2	0.36	0.047

#### KEGG enrichment

A total of 154 unique KEGG pathways were assigned to the 495 total DEGs (Table S1). Of the 320 salinity-related DEGs, 85 (26.6%) were assigned KO terms. Of the 364 rhizobia-related DEGs, 90 (24.7%) were assigned KO terms. Of the 179 DEGs expressed in the salinity x rhizobium interaction pool, 46 (25.7%) were assigned KO terms. Hypergeometric enrichment tests for salinity, rhizobia, and interaction DEGs returned no significantly enriched KEGG Orthology terms. For KEGG Orthology terms and the associated number of DEGs in a given DEG pool refer to Table S1.

The most common KEGG term within salinity-related DEGs was K17606 (annotated to 3 DEGs), coding for the TAP46 protein. Also, of note was K05016, coding for chloride channel 7 (2 DEGs), K09489 coding for the heat shock 70 kDA protein 4 (2 DEGs), and K09489 coding for the enhanced disease susceptibility 1 (EDS1) protein (2 DEGs). The most common KEGG terms among rhizobia-related DEGs (2 DEGs each) were K18875 (EDS1, enhanced disease susceptibility protein 1), K10523 (SPOP, speckled-type POZ protein), K17302 (COPB2/SEC27 protein coatomer subunit beta), K00966 (GMPP protein, mannose-1-phosphate guanylytransferase), K11253 (H3, histone H3), and K22520 (LQY1, protein disulfide isomerase).

There were no KEGG terms annotated to more than 1 DEG in the salinity x rhizobium interaction gene pool (refer to Table S1). K00830 (annotated to 1 DEG in the interaction pool) is associated with the glycine, serine, and threonine metabolism, similar to K02437. There were a total of 15 DEGs related to transcriptional or translational machinery that were significantly changed in rhizobium inoculated alfalfa at 8 dS/m salinity stress (Table 5).

**Table 5:** Transcriptional/translational related KEGG Ontology terms with gene identifiers, log2foldchange values, adjusted *p*-values, and brief protein descriptions

KO	Gene ID	baseMean	log_2_FC	P_adj_	Protein/ putative function
K17413	MS.gene08332.t1	51.42272546	30.0	< 0.001	MRPS35; small subunit ribosomal protein S35
K02996	MS.gene24329.t1	168.3939807	30.0	< 0.001	RP-S9, MRPS9, rpsI; small subunit ribosomal protein S9
K11593	MS.gene05149.t1	569.3381268	25.7	< 0.001	ELF2C, AGO; eukaryotic translation initiation factor 2C
K11253	MS.gene04748.t1	29.16856037	24.6	< 0.001	H3; histone H3
K02987	MS.gene74487.t1	143.8649843	24.5	< 0.001	RP-S4e, RPS4; small subunit ribosomal protein S4e
K11430	MS.gene07294.t1	212.1683553	22.3	< 0.001	EZH2; [histone H3]-lysine27 N-trimethyltransferase EZH2 [EC:2.1.1.356]
K14431	MS.gene88455.t1	158.5929093	21.2	< 0.001	TGA; transcription factor TGA
K22544	MS.gene79024.t1	130.9815078	21.0	< 0.001	SAMHD1; deoxynucleoside triphosphate triphosphohydrolase SAMHD1 [EC:3.1.5.-]
K01889	MS.gene23260.t1	69.81544634	15.6	< 0.001	FARSA, pheS; phenylalanyl-tRNA synthetase alpha chain [EC:6.1.1.20]
K15164	MS.gene75716.t1	156.9252144	-22.2	< 0.001	MED13; mediator of RNA polymerase II transcription subunit 13
K12875	MS.gene79245.t1	325.6574764	-23.1	< 0.001	ACIN1, ACINUS; apoptotic chromatin condensation inducer in the nucleus
K12823	MS.gene00082.t1	195.1528	-23.9	< 0.001	DDX5, DBP2; ATP-dependent RNA helicase DDX5/DBP2 [EC:3.6.4.13]
K02925	MS.gene59228.t1	711.1538539	-24.5	< 0.001	RP-L3e, RPL3; large subunit ribosomal protein L3e
K03252	MS.gene00590.t1	101.637428	-30.0	< 0.001	EIF3C; translation initiation factor 3 subunit C
K03093	MS.gene84140.t1	418.7448981	-30	< 0.001	sigI; RNA polymerase sigma factor

## Discussion

This experiment was designed to determine differential gene expression of salt-tolerant alfalfa stimulated by the rhizobium inoculant (*E. meliloti*) with or without salinity stress. This study detected a total of 495 DEGs related to salinity stress, inoculation, or inoculation x salinity stress. Many DEGs were related both to either salinity stress (320 DEGs) or rhizobium inoculation (364 DEGs), however fewer for the interaction between the two (179 DEGs). Of these DEGs, 78.4% of salinity-related DEGs, 71.4% of inoculant-related DEGs, and 76% of rhizobium x salinity interaction-related DEGs matched to GO terms, respectively. Of the 12 significantly enriched GO terms, 10 were associated with CC between salt-treated and non-salt treated plants; these GO terms all referenced membranes, vacuoles, and/or proton pumping. The remaining 2 were associated with BP (one related to salt vs non-salt treated plants and one related to the rhizobium x salinity interaction). The salinity-related term referenced the GO term “regulation of neurotransmitters” (NR/KEGG annotation “Glycine Cleavage System H protein”), while the interaction-related term referenced “sulfate assimilation”.

### Ion transport and storage related DEGs under salt stress

There were 11 enriched GO terms within salinity-related DEGs (Table 1). All of the enriched CC GO terms were associated with two genes; MS.gene 067465.t1 and MS.gene50737.t1. Furthermore, all of the 10 CC GO terms referenced cell membranes, intracellular organelles (including vacuoles), or membrane transporters, which highlights the importance of ion transport and storage in the salt tolerant alfalfa.

MS.gene50737.t1 was annotated to the GO term GO:0000220 (V-type ATPase) and the KEGG term K02154 (V-type H^+^ transporting ATPase sub-unit a). The V-type (V = vacuolar) transporting ATPase is an ATP powered H^+^ transporter which creates a proton gradient across membranes [29, 30]. V-type ATPases are important for the stress response of the plant [29], whereby they are capable aiding in the compartmentalization of toxic ions [31]. Various ATPases have been found to be associated with salinity tolerance of alfalfa in many previous transcriptomic studies [8, 10, 11, 24].

MS.gene067465.t1 was associated with GO:0043231 (intracellular membrane bounded organelle), which had no associated KEGG term. However, this gene was associated with the NR accession KEH39627, which codes for a SNAP receptor complex protein ( SNARE protein). SNARE proteins can be divided into v-type (vesicular) or t-type (target) and are responsible for facilitating vesicular transport between intracellular bodies [32]. Specific SNAREs have been shown to be active in transporting intracellular Na^+^ ions to the vacuole for compartmentalization [33], and have been identified as being upregulated in a previous transcriptomic study of wild rice (*Oryza australiensis*) when experiencing ionic stress [34]. Taken together, these results show an important role of vacuolar ion storage in the salinity tolerance of alfalfa.

In addition to vacuolar storage, 3 DEGs coding for ion channels were also upregulated in salt-stressed alfalfa. Two of these DEGs (MS.gene64059.t1, MS.gene74439.t1) coded for chloride channels (CLCN7, K05016), which are anion transporters responsible for transporting Cl^−^ and NO_3_^−^ [35], and one (MS.gene20294.t1) coded for a voltage dependant anion channel (VDAC2, K15040). An NR protein database cross-search returned separate terms for the CLC proteins: CLCN7 = CLC-a & CLC-c. CLC-a is a nitrate selective transporter while CLC-c is more chloride specific [36, 37]. CLCs are important for maintaining a healthy ratio of Cl^−^:NO_3_^−^, as excess Cl^−^ can impede nitrate assimilation [37, 38]. Both of the DEGs associated with CLCN7 were upregulated under 8 dS/m salinity stress, indicating that the salt-tolerant population used in this study had an intrinsic mechanism to attenuate chloride stress, thereby possibly increasing nitrate uptake. In our previous paper [14] we reported that nitrogen appeared to be a key factor in mediating salinity stress in salt-adapted alfalfa.

VDACs[1–5] are the major transport protein of the mitochondria and serve diverse functions, including transporting anions, accepting tRNA, ATP, and Ca^+^ ions, and mediating ROS gradients [39–41]. In this study, we found the transcripts of VDAC2 to increase in salt stressed plants. This is in contrast to recent work in *A. thaliana* and *Tritichum aestivum* L. where short term salt stress decreased transcription [42, 43]. The results of the current experiment suggest that after the potential decrease in expression noted by the previous studies, expression of VDAC2 may increase in alfalfa in response to a prolonged period of salinity stress.

### TOR, SNrK, and general stress response-related DEGs

Salt-adapted plants displayed upregulation of three transcripts related to the TAP42/TAP46 protein (MS.gene06912.t1, MS.gene06913.t1, and MS.gene49210.t1) as well as upregulation of a SNF1 (a.k.a SNrK, MS.gene75239.t1) protein. TAP42 is an important protein, which responds downstream to the TOR signalling pathway [44]. The TOR pathway, when active, promotes translation, anabolism, as well as nutrient uptake and metabolism of nitrogen. However, when TOR is deactivated by rapamycin, K^+^ deficiencies may be caused in addition to programmed cell death (PCD), and nutrient recycling [44, 45]. Research has shown Tap42- mutants to deactivate the TOR pathway and cause major downstream effects including reduced activity of nitrogen reductase (Nr) and other nitrogen metabolic genes [44, 45]. TOR’s close counterpart is the Sucrose Non-feRmenting Kinase (SNrK) pathway, controlled by the SNrk gene. The SNrK pathway is generally stimulated by the same factors which inhibit TOR [46]. The SNrK gene was downregulated in salt-stressed alfalfa. In contrast to salt-stressed plants, rhizobium-inoculated salt-stressed plants displayed upregulation of the SNrK1 gene and no change in the TAP42 gene, which suggests that growth of salt-tolerant alfalfa was inhibited by inoculation by *E. meliloti* under salt stress*.*

Interestingly, the AuTophaGy 1 gene (ATG1, MS.gene92581.t1), a downstream effector proteins of the TOR pathway, was also downregulated in salt-stressed plants. This protein is responsible for autophagy, and the ATG1 gene is modulated by the TOR complex, which in turn is stimulated in times of nitrogen starvation [47]. The fact that ATG1 was downregulated in salt-stressed vs control plants reinforces that the TOR complex was stimulated in salt-tolerant alfalfa.

There were 3 DEGs annotated to heat shock proteins, including HSP90 (K09487, MS.gene64023.t1) and HSPA4 a.k.a HSP70 (K09489, MS.gene33363.t1, MS.gene33365.t1). Both HSP90 and one of the HSP70 proteins were downregulated under salt stress, however this is congruent with the diverse nature of heat shock proteins. Heat shock proteins are not only metabolized in response to heat stress but are also produced or repressed in response to multiple other forms of abiotic stress including drought, cold, and salinity stress [48], and are reported by a previous transcriptomic study examining salt stress in alfalfa [10]. Previous work [48] reports that the upregulated HSP70 protein found in this study responds both to salinity and heat stress in plants. Interestingly, HSP70 may be transcriptionally regulated by a heat shock factor HSFA4, which also interacts with the SOS1 pathway to induce ion homeostasis under salt-stress [49].

### Disease resistance DEGs under salt stress

Similar to a previous study [11], this study found several immune-related genes modulated in response to salinity stress in alfalfa, including the relA, SPOP, SIAH1, and EDS1 genes. GTP pyrophosphokinase (relA, K00951, MS.gene26785.t1) was upregulated in salt stressed plants. This gene is also conserved in the chloroplasts of plants, and plays diverse roles in modulating transcription and translation to many stresses including pathogens and salinity [50]. Interestingly, the relA gene was upregulated under salinity stress, but downregulated in rhizobium inoculated, salt-stressed plants. The relA gene of the bacteroid, or its product ppGpp, have previously been shown to be important for the successful mutualism between legume and rhizobium [51–53]. It would be interesting for future studies to examine the root transcriptomics in rhizobium inoculated, salt-stressed alfalfa to determine the effects on relA transcription.

A speckle-type POZ protein (SPOP, K10523, MS.gene63029.t1), an E3-ubiquitin-protein ligase (SIAH1, K04506, MS.gene85969.t1), and two enhanced disease susceptibility protein (EDS1, K04506, MS.gene38404.t1, MS.gene38408.t1) genes were also upregulated in salt-stressed vs control plants. The SPOP gene codes for production of the Broad complex-Tramway-Bric-a-brac (BTB)-POZ protein, which gives specificity to the Cul3 Cullin-Ring E3 ligases (the E3-ligase upregulated in this study) when ubiquinating cellular compounds for disposal [54–57]. Interestingly, E3-ligases were also implicated in the salt-stress response of alfalfa in previous transcriptomic studies examining salt-stressed alfalfa [8, 26, 27]. One of the functions of this ligase is to mediate levels of the EDS1 protein, upregulated in this study. EDS1 is a central protein to much of plants’ immune-response [58, 59], however research has also suggested that it is required for the functioning of specific drought-response genes in *Arabidopsis thaliana* [60]*.* The upregulation of the various components of the EDS1 pathway, in addition to corroboration by a previous transcriptomic study, suggests an important role of the pathway in mediation of salt-stress in alfalfa.

### Effect of rhizobium inoculation on salt-stressed plants

The results of the differential expression analysis showed that there were 179 genes which were differentially expressed in rhizobium inoculated, salt-stressed plants. Of these 179 genes, 76 were upregulated and 103 were downregulated. Inoculation of salt-stressed alfalfa induced 46 genes with known KEGG proteins. Several of these were directly or indirectly associated with salinity tolerance in relation to epigenetics, nutrient uptake, osmoprotectants, or photorespiration.

Fifteen DEGs related to transcriptional, translational, or other forms of genetic infrastructure in the host plants were modulated in inoculated, salt-stressed plants (see Table 5). These included a DEAD box RNA helicase (DDX5, K12823, MS.gene00082.t1), an argonaute protein (AGO, K11593, MS.gene05149.t1), and several genes related to epigenetic control (EZH2, K11430, MS.gene07294.t1 & H3, K11253, MS.gene04748.t1). Of these proteins, DEAD box helicases have previously been shown to mediate salt stress in *A. thaliana* by increasing activity of ROS scavengers and stimulating production of the osmoprotectant proline [61], which was a key finding in our original work [14]. An upregulated RNA helicase gene has also been previously reported in salt-tolerant alfalfa [8], and a previous review [62] reports many studies in which chloroplastic/mitochondrial DEAD box helicases mediate various abiotic stresses.

The argonaute (AGO) gene was also upregulated in inoculated/salt-stressed plants. argonaute is the housing protein into which micro-RNAs (miRNA) are housed to create an miRNA-Induced-Silencing Complex miRISC; miRISCs have been implicated in plant responses to many stresses whereby they silence translation of specific mRNA transcripts [63], and non-coding RNAs (of which miRNAs are a subset) are reported in previous transcriptomic work with salt-stressed alfalfa [24]. The fact that AGO was upregulated in inoculated/salt-stressed plants indicates that post-transcriptional control was taking place to modify the expression of potentially stress-related genes. Interestingly, assembly of the miRISC complex requires HSP90 [63], another DEG found in this study.

Perhaps the most interesting effects were the upregulation of both the histone H3 (H3, MS.gene04748.t1) as well as the lysine 27 N-trimethyltransferase (EZH2 a.k.a H3K27me3, MS.gene07294.t1), both related to epigenetics. Five variants of histones work together to form a nucleosome in combination with DNA [64]: H2A, H2B, H3, and H4 (with H5 serving as a link histone [65]). DNA is wrapped around these histones for storage but unwound for transcription. Histones can be methylated, acetylated, or phosphorylated at their N-terminus (tail end) to promote or preclude transcription, causing epigenetic control of genes [66]. Recent work with Castor bean (*Ricinus communis*) under salinity stress [67] has shown that the EZH2 (a.k.a H3K27me3) enzyme exerts epigenetic control over salt-stress related genes. The authors report that the addition of H3K27me3 to histones was correlated with DEG repression, while its loss was correlated with normal transcription. Another study [68] showed that removal of H3K27me3 from the High Affinity Potassium transporter 1 (HKT1) gene in *A. thaliana* induced an improved ability to mediate salt stress in future events. Previous transcriptomic studies examining salt-stressed alfalfa have also reported epigenetic-related methyltransferases/demethylases [10, 11]. Very interestingly in this study, the EZH2 DEG was significantly downregulated in inoculated plants whereas inoculated/salt-stressed plants experienced a large-scale increase in transcription of EZH2. This result matches another recent transcriptomic study which also found upregulation of a H3-lysine methyltransferase in salt-tolerant alfalfa [8].

The significant upregulation of H3K27me3 under inoculated-saline conditions in this study clearly indicates that *E. meliloti* is associated with inhibition of gene transcription for stress-related genes in alfalfa. As the addition/removal of H3K27me3 can prime plants for future stress, understanding the relationship between this bacteria and plant transcription could serve to protect plants from future stress events. Additionally, the overlap between breeding and epigenetics is beginning to receive attention (i.e., [69]), and future study may benefit from examining the effects of mutualistic-bacterial inoculation on this phenomenon, as bacteria could serve as a mechanism to repress/de-repress transcription of important genes.

### DEGs for nutrient uptake under salt stress

The Adenosine-5-phosphosulfate reductase (APS reductase, K13811, MS.gene006381.t1) and Fe-S Cluster Assembly ATP-binding protein (sufC, K09013, MS.gene55865.t1) genes related to sulfur metabolism were upregulated in rhizobium inoculated, salt-stressed plants. The APS reductase gene was annotated by the enriched GO term GO:0000103 (“sulfate assimilation”). APS reductase is one of the key enzymes controlling the assimilation of sulfate, and the production of the plants first sulfur containing product: cysteine [70]. The APS reductase enzyme requires an Fe-S cluster as a cofactor [71], and the sufC gene also upregulated in this study, is at least partially responsible for synthesizing Fe-S clusters [72]. Sulfur metabolism, with cysteine being the organic building block, is vital for the stress response of plants as the important stress-signalling hormones abscisic acid, salicylate, and jasmonate in addition to the ROS scavenger glutathione are all sulfur based [73, 74]. However, we did not discover significant DEGs related to N update under stress.

### Osmoprotectants and photorespiration related DEGs

The Xaa-Pro peptidase (pepP, K01262, MS.gene89849.t1) gene related to the osmoprotectant proline, alpha-galactosidase (galA, K07407, MS.gene87685.t1) gene responsible for galactinol/myo-inositol conversions, and a gene related to glycine (AGXT, K00830, MS.gene92042.t1) production were all downregulated in inoculated/salt-stressed plants. pepP proteins can act to cleaving off any amino acid off of the N-terminal of a peptide that is attached to a proline element [75], which can prepare proteins with an N-terminal proline to be acted upon by a proline aminopeptidase (PAP), which have been shown to increase proline levels in response to abiotic stress [76]. Proline acts both to combat ROS accumulation and to stabilize osmotic potential across membranes [77, 78]. In contrast to proline, the galA gene is responsible for myo-inositol/galactinol interconversions; myo-inositol is an important osmoprotectant while galactinol may serve as an ROS scavenger [79, 80]. Modulation of beta-galactosidase and inositol transporter genes have been implicated by previous transcriptomic studies of salt-stressed alfalfa [10, 11]. Another interesting result was the downregulation of the AGXT gene. AGXT directly governs the reversible reaction creating glycine (a vital componenet of the osmoprotectant glycine betaine) from serine glyoxylate [81]. As these enzymes catalyzed reversible reactions, the effect on the pool of osmoprotectants is unknown.

Another interesting result was the down regulation of the glycine cleavage proteins in inoculated/salt-stressed plants, which also acts upon the compound glycine. The glycine cleavage proteins are part of the glyoxylate decarboxylase complex (GDC) which is composed of glycine cleavage proteins P, L, T, and H. gcvH (K02437, MS.gene21474.t1) was strongly downregulated in inoculated/salt-stressed plants. The GDC complex is responsible for creating serine from glycine in a two-step reaction within the mitochondria [82], an important step in the photorespiratory pathway in which phosphoglycolate is recycled into 3-phosphoglycerate after oxygen is mistaken for CO_2_ by the rubisco enzyme. Salt-stressed vs control plants in this trial exhibited elevated expression of the gcvH complex, however expression in inoculated/salt-stressed plants was depressed showing that bacteria were associated with a strong effect on transcription of this gene. Recent studies [83, 84] clearly demonstrate that mutualistic bacteria can reduce the rates of photorespiration in salt-stressed crops by overexpressing photorespiratory enzymes, including elements of the glycine cleavage system.

## Conclusion

Altogether, results of this study show that salt-tolerant alfalfa responded to 8 dS/m salinity by increasing production of ion transporters and by repressing PCD and nitrogen catabolism through a promoted TOR and inhibited SNrK pathway. *E. meliloti* caused both advantageous and deleterious changes to gene expression levels of salt-stressed alfalfa; these included stimulating sulfate assimilation through upregulation of APS reductase, but also downregulating genes responsible for creating more free proline (pepP) and effective photorespiration (gcvH). An ambiguous result which may deserve future research was the differential expression of genes related to epigenetics and post-transcriptional control of genes, including the EZH2 and AGO genes seen in inoculated/salt-stressed plants. The effect on these two genes offers a tantalizing glimpse at the complex genetic relationship between salt stress, legumes, and rhizobium.

### Supplementary Information

Supplementary Material 1.

## Data Availability

NCBI SRA Database, Accession PRJNA1071613.

## Abbreviations

KEGG: Kyoto Encyclopedia of Genes and Genomes
RNA-seq: RNA Sequencing
GO: Gene Ontology
NR: NCBI Non-Redundant Protein Database
TOR: Target of Rapamycin
SNrK: Sucrose Non Fermenting Kinase
EZH2: Histone H3 Lysine-27-Trimethyltransferase
AGO: Argonaute
CLC: Chloride Channel
VDAC: Voltage Dependent Anion Channel
TAP42: Type 2A Phosphatase Associated Protein 42Kd
PP2A: Protein Phosphatase 2A
ATG1: Autophagy 1
ppGpp: Guanosine Tetraphosphate
SPOP: Speckle type POZ Protein
EDS1: Enhanceded Disease Susceptibility 1
HSP: Heat Shock Protein
pepP: Proline Peptidase
AGXT: Alanine Glyoxylate Transaminase
gcvH: Glycine Cleavage system H protein
GDC: Glyoxylate Decarboxylase Complex
