# Supplementary material for: Differential gene expression of salt-tolerant alfalfa in response to salinity and inoculation by Ensifer meliloti
Source: BMC Plant Biol. 2024 Jul 6;24:633. doi: 10.1186/s12870-024-05337-5 (PMC11227210; doi:10.1186/s12870-024-05337-5)
Supplement: Supplementary file 1 — Supplementary Material 1. [file 12870_2024_5337_MOESM1_ESM.docx]

Table S1. Number of DEGs annotated to KEGG orthology terms.

|  |  |  |  |  |
| --- | --- | --- | --- | --- |
| KOID | Salinity | Bac | Interaction | KEGG Term |
| K17606 | 3 | 0 | 0 | IGBP1, TAP42; immunoglobulin-binding protein 1 |
| K11593 | 2 | 0 | 1 | ELF2C, AGO; eukaryotic translation initiation factor 2C |
| K18875 | 2 | 2 | 0 | EDS1; enhanced disease susceptibility 1 protein |
| K05016 | 2 | 1 | 0 | CLCN7; chloride channel 7 |
| K09489 | 2 | 0 | 0 | HSPA4; heat shock 70 kDa protein 4 |
| K17756 | 2 | 0 | 0 | FAO3; long-chain-alcohol oxidase [EC:1.1.3.20] |
| K10523 | 1 | 2 | 1 | SPOP; speckle-type POZ protein |
| K00815 | 1 | 1 | 1 | TAT; tyrosine aminotransferase [EC:2.6.1.5] |
| K00951 | 1 | 1 | 1 | relA; GTP pyrophosphokinase [EC:2.7.6.5] |
| K00975 | 1 | 1 | 1 | glgC; glucose-1-phosphate adenylyltransferase [EC:2.7.7.27] |
| K01262 | 1 | 1 | 1 | pepP; Xaa-Pro aminopeptidase [EC:3.4.11.9] |
| K02437 | 1 | 1 | 1 | gcvH, GCSH; glycine cleavage system H protein |
| K08917 | 1 | 1 | 1 | LHCB6; light-harvesting complex II chlorophyll a/b binding protein 6 |
| K14684 | 1 | 1 | 1 | SLC25A23S; solute carrier family 25 (mitochondrial phosphate transporter),  member 23/24/25/41 |
| K15040 | 1 | 1 | 1 | VDAC2; voltage-dependent anion channel protein 2 |
| K15042 | 1 | 1 | 1 | KPNA5_6; importin subunit alpha-6/7 |
| K15283 | 1 | 1 | 1 | SLC35E1; solute carrier family 35, member E1 |
| K23544 | 1 | 1 | 1 | SERINC1; serine incorporator 1 |
| K02987 | 1 | 0 | 1 | RP-S4e, RPS4; small subunit ribosomal protein S4e |
| K05665 | 1 | 0 | 1 | ABCC1; ATP-binding cassette, subfamily C (CFTR/MRP), member 1 [EC:7.6.2.3] |
| K09013 | 1 | 0 | 1 | sufC; Fe-S cluster assembly ATP-binding protein |
| K14431 | 1 | 0 | 1 | TGA; transcription factor TGA |
| K17302 | 1 | 2 | 0 | COPB2, SEC27; coatomer subunit beta' |
| K00801 | 1 | 1 | 0 | FDFT1; farnesyl-diphosphate farnesyltransferase [EC:2.5.1.21] |
| K01723 | 1 | 1 | 0 | AOS; hydroperoxide dehydratase [EC:4.2.1.92] |
| K01726 | 1 | 1 | 0 | GAMMACA; gamma-carbonic anhydrase [EC:4.2.1.-] |
| K02726 | 1 | 1 | 0 | PSMA2; 20S proteasome subunit alpha 2 [EC:3.4.25.1] |
| K03260 | 1 | 1 | 0 | EIF4G; translation initiation factor 4G |
| K03768 | 1 | 1 | 0 | PPIB, ppiB; peptidyl-prolyl cis-trans isomerase B (cyclophilin B) [EC:5.2.1.8] |
| K06617 | 1 | 1 | 0 | E2.4.1.82; raffinose synthase [EC:2.4.1.82] |
| K06630 | 1 | 1 | 0 | YWHAE; 14-3-3 protein epsilon |
| K09667 | 1 | 1 | 0 | OGT; protein O-GlcNAc transferase [EC:2.4.1.255] |
| K11843 | 1 | 1 | 0 | USP14, UBP6; ubiquitin carboxyl-terminal hydrolase 14 [EC:3.4.19.12] |
| K13457 | 1 | 1 | 0 | RPM1, RPS3; disease resistance protein RPM1 |
| K14326 | 1 | 1 | 0 | UPF1, RENT1; regulator of nonsense transcripts 1 [EC:3.6.4.13 5.6.2.3] |
| K14682 | 1 | 1 | 0 | argAB; amino-acid N-acetyltransferase [EC:2.3.1.1] |
| K15210 | 1 | 1 | 0 | SNAPC3; snRNA-activating protein complex subunit 3 |
| K17108 | 1 | 1 | 0 | GBA2; non-lysosomal glucosylceramidase [EC:3.2.1.45] |
| K17873 | 1 | 1 | 0 | CYP72A; 11-oxo-beta-amyrin 30-oxidase [EC:1.14.14.115] |
| K20223 | 1 | 1 | 0 | IPO7, RANBP7; importin-7 |
| K00454 | 1 | 0 | 0 | LOX2S; lipoxygenase [EC:1.13.11.12] |
| K00522 | 1 | 0 | 0 | FTH1; ferritin heavy chain [EC:1.16.3.2] |
| K00703 | 1 | 0 | 0 | glgA; starch synthase [EC:2.4.1.21] |
| K00819 | 1 | 0 | 0 | rocD, OAT; ornithine-oxo-acid transaminase [EC:2.6.1.13] |
| K01051 | 1 | 0 | 0 | E3.1.1.11; pectinesterase [EC:3.1.1.11] |
| K01369 | 1 | 0 | 0 | LGMN; legumain [EC:3.4.22.34] |
| K01535 | 1 | 0 | 0 | PMA1, PMA2; H+-transporting ATPase [EC:7.1.2.1] |
| K02154 | 1 | 0 | 0 | ATPeV0A, ATP6N; V-type H^+^ transporting ATPase subunit a |
| K02183 | 1 | 0 | 0 | CALM; calmodulin |
| K03066 | 1 | 0 | 0 | PSMC5, RPT6; 26S proteasome regulatory subunit T6 |
| K03347 | 1 | 0 | 0 | CUL1, CDC53; cullin 1 |
| K03353 | 1 | 0 | 0 | APC6, CDC16; anaphase-promoting complex subunit 6 |
| K03686 | 1 | 0 | 0 | dnaJ; molecular chaperone DnaJ |
| K04506 | 1 | 0 | 0 | SIAH1; E3 ubiquitin-protein ligase SIAH1 [EC:2.3.2.27] |
| K07374 | 1 | 0 | 0 | TUBA; tubulin alpha |
| K08269 | 1 | 0 | 0 | ULK2, ATG1; serine/threonine-protein kinase ULK2 [EC:2.7.11.1] |
| K08288 | 1 | 0 | 0 | PRKCSH; protein kinase C substrate 80K-H |
| K08516 | 1 | 0 | 0 | YKT6; synaptobrevin homolog YKT6 |
| K08679 | 1 | 0 | 0 | GAE, cap1J; UDP-glucuronate 4-epimerase [EC:5.1.3.6] |
| K08905 | 1 | 0 | 0 | psaG; photosystem I subunit V |
| K09487 | 1 | 0 | 0 | HSP90B, TRA1; heat shock protein 90kDa beta |
| K10255 | 1 | 0 | 0 | FAD6, desA; acyl-lipid omega-6 desaturase (Delta-12 desaturase) [EC:1.14.19.23 1.14.19.45] |
| K10400 | 1 | 0 | 0 | KIF15; kinesin family member 15 |
| K10579 | 1 | 0 | 0 | UBE2M, UBC12; ubiquitin-conjugating enzyme E2 M [EC:2.3.2.34] |
| K10610 | 1 | 0 | 0 | DDB1; DNA damage-binding protein 1 |
| K12449 | 1 | 0 | 0 | AXS; UDP-apiose/xylose synthase |
| K12761 | 1 | 0 | 0 | SNF1; carbon catabolite-derepressing protein kinase [EC:2.7.11.1] |
| K12879 | 1 | 0 | 0 | THOC2; THO complex subunit 2 |
| K12897 | 1 | 0 | 0 | TRA2; transformer-2 protein |
| K14484 | 1 | 0 | 0 | IAA; auxin-responsive protein IAA |
| K14514 | 1 | 0 | 0 | EIN3; ethylene-insensitive protein 3 |
| K14567 | 1 | 0 | 0 | UTP14; U3 small nucleolar RNA-associated protein 14 |
| K16278 | 1 | 0 | 0 | HOS1; E3 ubiquitin-protein ligase HOS1 [EC:2.3.2.27] |
| K17267 | 1 | 0 | 0 | COPG; coatomer subunit gamma |
| K17398 | 1 | 0 | 0 | DNMT3A; DNA (cytosine-5)-methyltransferase 3A [EC:2.1.1.37] |
| K17982 | 1 | 0 | 0 | TPS04, GES; geranyllinalool synthase [EC:4.2.3.144] |
| K21989 | 1 | 0 | 0 | TMEM63, CSC1; calcium permeable stress-gated cation channel |
| K22503 | 1 | 0 | 0 | DARS1; aspartyl-tRNA synthetase [EC:6.1.1.12] |
| K00966 | 0 | 2 | 1 | GMPP; mannose-1-phosphate guanylyltransferase [EC:2.7.7.13] |
| K11253 | 0 | 2 | 1 | H3; histone H3 |
| K01904 | 0 | 1 | 1 | 4CL; 4-coumarate-CoA ligase [EC:6.2.1.12] |
| K11430 | 0 | 1 | 1 | EZH2; [histone H3]-lysine27 N-trimethyltransferase EZH2 [EC:2.1.1.356] |
| K17872 | 0 | 1 | 1 | NDC1, ndbB; demethylphylloquinone reductase [EC:1.6.5.12] |
| K00830 | 0 | 0 | 1 | AGXT; alanine-glyoxylate transaminase / serine-glyoxylate transaminase/  serine-pyruvate transaminase [EC:2.6.1.44 2.6.1.45 2.6.1.51] |
| K01338 | 0 | 0 | 1 | lon; ATP-dependent Lon protease [EC:3.4.21.53] |
| K01537 | 0 | 0 | 1 | ATP2C; P-type Ca2+ transporter type 2C [EC:7.2.2.10] |
| K01889 | 0 | 0 | 1 | FARSA, pheS; phenylalanyl-tRNA synthetase alpha chain [EC:6.1.1.20] |
| K02925 | 0 | 0 | 1 | RP-L3e, RPL3; large subunit ribosomal protein L3e |
| K02996 | 0 | 0 | 1 | RP-S9, MRPS9, rpsI; small subunit ribosomal protein S9 |
| K03093 | 0 | 0 | 1 | sigI; RNA polymerase sigma factor |
| K03252 | 0 | 0 | 1 | EIF3C; translation initiation factor 3 subunit C |
| K07198 | 0 | 0 | 1 | PRKAA, AMPK; 5'-AMP-activated protein kinase, catalytic alpha subunit [EC:2.7.11.11] |
| K07407 | 0 | 0 | 1 | E3.2.1.22B, galA, rafA; alpha-galactosidase [EC:3.2.1.22] |
| K08360 | 0 | 0 | 1 | CYB561; transmembrane ascorbate-dependent reductase [EC:7.2.1.3] |
| K08511 | 0 | 0 | 1 | ATVAMP72; vesicle-associated membrane protein 72 |
| K08869 | 0 | 0 | 1 | ADCK, ABC1; aarF domain-containing kinase |
| K12823 | 0 | 0 | 1 | DDX5, DBP2; ATP-dependent RNA helicase DDX5/DBP2 [EC:3.6.4.13] |
| K12875 | 0 | 0 | 1 | ACIN1, ACINUS; apoptotic chromatin condensation inducer in the nucleus |
| K12890 | 0 | 0 | 1 | SRSF1, SFRS1, ASF, SF2; serine/arginine-rich splicing factor 1 |
| K14006 | 0 | 0 | 1 | SEC23; protein transport protein SEC23 |
| K14652 | 0 | 0 | 1 | ribBA; 3,4-dihydroxy 2-butanone 4-phosphate synthase /  GTP cyclohydrolase II [EC:4.1.99.12 3.5.4.25] |
| K15164 | 0 | 0 | 1 | MED13; mediator of RNA polymerase II transcription subunit 13 |
| K15891 | 0 | 0 | 1 | FLDH; NAD^+^-dependent farnesol dehydrogenase [EC:1.1.1.354] |
| K17413 | 0 | 0 | 1 | MRPS35; small subunit ribosomal protein S35 |
| K18667 | 0 | 0 | 1 | ASCC2; activating signal cointegrator complex subunit 2 |
| K19269 | 0 | 0 | 1 | PGP, PGLP; phosphoglycolate phosphatase [EC:3.1.3.18 3.1.3.48] |
| K22544 | 0 | 0 | 1 | SAMHD1; deoxynucleoside triphosphate triphosphohydrolase SAMHD1 [EC:3.1.5.-] |
| K22520 | 0 | 2 | 0 | LQY1; protein disulfide-isomerase [EC:5.3.4.1] |
| K00344 | 0 | 1 | 0 | qor, CRYZ; NADPH:quinone reductase [EC:1.6.5.5] |
| K00558 | 0 | 1 | 0 | DNMT1, dcm; DNA (cytosine-5)-methyltransferase 1 [EC:2.1.1.37] |
| K00615 | 0 | 1 | 0 | E2.2.1.1, tktA, tktB; transketolase [EC:2.2.1.1] |
| K00799 | 0 | 1 | 0 | GST, gst; glutathione S-transferase [EC:2.5.1.18] |
| K00889 | 0 | 1 | 0 | PIP5K; 1-phosphatidylinositol-4-phosphate 5-kinase [EC:2.7.1.68] |
| K01061 | 0 | 1 | 0 | E3.1.1.45; carboxymethylenebutenolidase [EC:3.1.1.45] |
| K01183 | 0 | 1 | 0 | E3.2.1.14; chitinase [EC:3.2.1.14] |
| K01251 | 0 | 1 | 0 | AHCY, ahcY; adenosylhomocysteinase [EC:3.13.2.1] |
| K01366 | 0 | 1 | 0 | CTSH; cathepsin H [EC:3.4.22.16] |
| K01373 | 0 | 1 | 0 | CTSF; cathepsin F [EC:3.4.22.41] |
| K02937 | 0 | 1 | 0 | RP-L7e, RPL7; large subunit ribosomal protein L7e |
| K03083 | 0 | 1 | 0 | GSK3B; glycogen synthase kinase 3 beta [EC:2.7.11.26] |
| K05349 | 0 | 1 | 0 | bglX; beta-glucosidase [EC:3.2.1.21] |
| K06013 | 0 | 1 | 0 | STE24; STE24 endopeptidase [EC:3.4.24.84] |
| K06067 | 0 | 1 | 0 | HDAC1_2; histone deacetylase 1/2 [EC:3.5.1.98] |
| K08081 | 0 | 1 | 0 | TR1; tropinone reductase I [EC:1.1.1.206] |
| K08675 | 0 | 1 | 0 | PRSS15, PIM1; ATP-dependent Lon protease [EC:3.4.21.53] |
| K08867 | 0 | 1 | 0 | WNK, PRKWNK; WNK lysine deficient protein kinase [EC:2.7.11.1] |
| K08915 | 0 | 1 | 0 | LHCB4; light-harvesting complex II chlorophyll a/b binding protein 4 |
| K08955 | 0 | 1 | 0 | YME1; ATP-dependent metalloprotease [EC:3.4.24.-] |
| K09874 | 0 | 1 | 0 | NIP; aquaporin NIP |
| K10355 | 0 | 1 | 0 | ACTF; actin, other eukaryote |
| K10406 | 0 | 1 | 0 | KIFC2_3; kinesin family member C2/C3 |
| K10664 | 0 | 1 | 0 | ATL6S; E3 ubiquitin-protein ligase ATL6/9/15/31/42/55 [EC:2.3.2.27] |
| K10839 | 0 | 1 | 0 | RAD23, HR23; UV excision repair protein RAD23 |
| K11275 | 0 | 1 | 0 | H1_5; histone H1/5 |
| K12349 | 0 | 1 | 0 | ASAH2; neutral ceramidase [EC:3.5.1.23] |
| K12483 | 0 | 1 | 0 | EHD1; EH domain-containing protein 1 |
| K12580 | 0 | 1 | 0 | CNOT3, NOT3; CCR4-NOT transcription complex subunit 3 |
| K12619 | 0 | 1 | 0 | XRN2, RAT1; 5'-3' exoribonuclease 2 [EC:3.1.13.-] |
| K12844 | 0 | 1 | 0 | PRPF31; U4/U6 small nuclear ribonucleoprotein PRP31 |
| K13336 | 0 | 1 | 0 | PEX3; peroxin-3 |
| K13418 | 0 | 1 | 0 | SERK1; somatic embryogenesis receptor kinase 1 [EC:2.7.10.1 2.7.11.1] |
| K13947 | 0 | 1 | 0 | PIN; auxin efflux carrier family protein |
| K14545 | 0 | 1 | 0 | RRP7; ribosomal RNA-processing protein 7 |
| K14818 | 0 | 1 | 0 | SQT1; ribosome assembly protein SQT1 |
| K15403 | 0 | 1 | 0 | ACE, HTH; fatty acid omega-hydroxy dehydrogenase [EC:1.1.-.-] |
| K15692 | 0 | 1 | 0 | RNF13, RZF; E3 ubiquitin-protein ligase RNF13 [EC:2.3.2.27] |
| K16075 | 0 | 1 | 0 | MRS2, MFM1; magnesium transporter |
| K16571 | 0 | 1 | 0 | TUBGCP4, GCP4; gamma-tubulin complex component 4 |
| K18443 | 0 | 1 | 0 | GBF1; golgi-specific brefeldin A-resistance guanine nucleotide exchange factor 1 |
| K18810 | 0 | 1 | 0 | CYCD1_2_4; cyclin D1/2/4, plant |
| K20473 | 0 | 1 | 0 | NBAS; neuroblastoma-amplified sequence |
| K20628 | 0 | 1 | 0 | exlX; expansin |
| K21444 | 0 | 1 | 0 | PCBP3_4; poly(rC)-binding protein 3/4 |
| K21596 | 0 | 1 | 0 | CAMTA; calmodulin-binding transcription activator |
